# Survey of Obstetrician-Gynecologists in the United States About Toxoplasmosis

**DOI:** 10.1155/S1064744901000059

**Published:** 2001

**Authors:** Jeffrey L. Jones, Vance J. Dietz, Michael Power, Adriana Lopez, Marianna Wilson, Thomas R. Navin, Ronald Gibbs, Jay Schulkin

**Affiliations:** 1 Mailstop F-22 Division of Parasitic Diseases Centers for Disease Control and Prevention 4770 Buford Highway NE Atlanta GA 30341-3724 USA; 2 American College of Obstetricians and Gynecologists Washington, DC USA; 3 University of Colorado Health Sciences Center Denver CO USA

## Abstract

*Background:* Although the incidence of toxoplasmosis is low in the United States, up to 6000 congenital cases occur annually. In September 1998, the Centers for Disease Control and Prevention held a conference about toxoplasmosis; participants recommended a survey of the toxoplasmosis-related knowledge and practices of obstetrician-gynecologists and the development of professional educational materials for them.

*Methods:* In the fall of 1999, surveys were mailed to a 2% random sample of American College of Obstetricians and Gynecologists (ACOG) members and to a demographically representative group of ACOGmembers known as the *Collaborative Ambulatory Research Network* (CARN). Responses were not significantly different for the random and CARN groups for most questions (*p* value shown when different).

*Results:* Among 768 US practicing ACOG members surveyed, 364 (47%) responded. Seven per cent (CARN 10%, random 5%) had diagnosed one or more case(s) of acute toxoplasmosis in the past year. Respondents were well-informed about how to prevent toxoplasmosis. However, only 12% (CARN 11%, random 12%) indicated that a positive *Toxoplasma* IgM test might be a false–positive result, and only 11% (CARN 14%, random 9%) were aware that the Food and Drug Administration sent an advisory to all ACOG members in 1997 stating that some *Toxoplasma* IgM test kits have high false–positive rates. Most of those surveyed (CARN 70%, random 59%; X^2^ *p* < 0.05) were opposed to universal screening of pregnant women.

*Conclusions:* Many US obstetrician-gynecologists will encounter acute toxoplasmosis during their careers, but they are frequently uncertain about interpretation of the laboratory tests for the disease. Most would not recommend universal screening of pregnant women.


Infect Dis Obstet Gynecol 2001;9:23–31

### Survey of obstetrician-gynecologists in the United States about toxoplasmosis

**Jeffrey L. Jones[1], Vance J. Dietz[1], Michael Power[2], Adriana Lopez[1], Marianna Wilson[1], Thomas R. Navin[1], Ronald Gibbs[3] and Jay Schulkin[2]**

[1]*Centers for Disease Control and Prevention, Atlanta, GA*
[2]*American College of Obstetricians and Gynecologists, Washington, DC*
[3]*University of Colorado Health Sciences Center, Denver, CO*

***Background:*** Although the incidence of toxoplasmosis is low in the United States, up to 6000 congenital cases occur annually. In September 1998, the Centers for Disease Control and Prevention held a conference about toxoplasmosis; participants recommended a survey of the toxoplasmosis-related knowledge and practices of obstetrician-gynecologists and the development of professional educational materials for them.
***Methods:*** In the fall of 1999, surveys were mailed to a 2% random sample of American College of Obstetricians and Gynecologists (ACOG) members and to a demographically representative group of ACOG members known as the *Collaborative Ambulatory Research Network* (CARN). Responses were not significantly different for the random and CARN groups for most questions (*p* value shown when different).
***Results:*** Among 768 US practicing ACOG members surveyed, 364 (47%) responded. Seven per cent (CARN 10%, random 5%) had diagnosed one or more case(s) of acute toxoplasmosis in the past year. Respondents were well-informed about how to prevent toxoplasmosis. However, only 12% (CARN 11%, random 12%) indicated that a positive *Toxoplasma* IgM test might be a false–positive result, and only 11% (CARN 14%, random 9%) were aware that the Food and Drug Administration sent an advisory to all ACOG members in 1997 stating that some *Toxoplasma* IgM test kits have high false–positive rates. Most of those surveyed (CARN 70%, random 59%; $\chi^2$ $p < 0.05$) were opposed to universal screening of pregnant women.
***Conclusions:*** Many US obstetrician-gynecologists will encounter acute toxoplasmosis during their careers, but they are frequently uncertain about interpretation of the laboratory tests for the disease. Most would not recommend universal screening of pregnant women.

Key words: *TOXOPLASMA GONDII*; PHYSICIAN KNOWLEDGE

Although toxoplasmosis has a relatively low incidence in the United States, it is still the third leading cause of food-borne deaths[1], and estimates range from 400 to 6000 congenital infections per year[1–4], which can lead to impaired vision, hearing deficits and mental retardation. Obstetrician-gynecologists may be confronted with a number of issues regarding toxoplasmosis, including when to test or screen pregnant women, how to interpret laboratory test results, and how to manage patients clinically. In September 1998, the Centers for Disease Control and Prevention (CDC) held a

Correspondence to: Dr Jeffrey L. Jones, Mailstop F-22, Division of Parasitic Diseases, Centers for Disease Control and Prevention, 4770 Buford Highway NE, Atlanta, GA 30341-3724, USA. E-mail: JLJ1@CDC.gov conference about toxoplasmosis to identify research and prevention priorities[4]. Participants attended from governmental, medical, academic and industrial institutions. One of the recommendations of this conference was to determine the toxoplasmosis-related knowledge and practices of obstetrician-gynecologists and to develop professional education materials tailored to their needs. As a result of this recommendation, the CDC and the Department of Research of the American College of Obstetrician and Gynecologists (ACOG) collaborated to survey obstetrician-gynecologists in the United States.

### SUBJECTS AND METHODS

A survey instrument was developed by the Department of Research of ACOG and CDC to collect information from obstetrician-gynecologists about their practice and patient characteristics, knowledge about laboratory diagnostic tests, laboratory screening and testing practices, knowledge about clinical toxoplasmosis and prevention of infection, and preventive counseling practices. Questions were primarily in the multiple-choice format. Input for development of the survey was obtained from obstetrician-gynecologists, laboratory workers, epidemiologists, public health physicians, and infectious disease physicians.

In the fall of 1999, the six-page questionnaire was sent by the Department of Research of ACOG to a 2% random sample of ACOG members ($n = 786$) and to another group of ACOG members known as the Collaborative Ambulatory Research Network (CARN; $n = 224$). The members of the network are practicing obstetrician-gynecologists chosen to be demographically representative of ACOG members by age and sex who voluntarily participate in surveys made periodically by the college. A second mailing of the questionnaire was made to nonrespondents 3 weeks after the first. Questionnaires were compiled and data entered with a unique ID number at ACOG headquarters in Washington, DC. Only respondents that practiced in the United States were included in the analysis.

Response proportions for the random and CARN groups were compared by the $\chi^2$ test and footnoted when the difference was statistically sigificant ($p < 0.05$; see footnote in Table 2). Combined responses only are given in the text unless: (1) the CARN and random group responses were statistically different, or (2) data are presented in the text but not in Table 2. In these two cases the CARN and random responses are also given in the text. Ninety-five per cent confidence intervals were calculated with StatXact using the Clopper–Pearson method[5]. Variances were similar for the CARN and random group responses by the F-test, so 95% confidence limits were calculated for the combined groups. Data analyses were performed with EpiInfo[6] and SAS[7] software, except for responses to open-ended questions that were categorized and tabulated by hand. Because there were so many types of responses, the data for the open-ended questions are summarized in the text but are not shown in Table 2. Student's *t* test was used to compare the means for some of the demographic characteristics. Percentages are rounded to the nearest 1% in the text and to the nearest 0.1% in the tables.

### RESULTS

### Response rate

Among the 224 CARN obstetrician-gynecologists surveyed who practiced in the United States, 164 (73%) responded. Among the 786 randomly selected obstetrician-gynecologists practicing in the United States, 296 (38%) responded. Ninety-six persons returned the questionnaire without responding because they did not see obstetric patients, leaving 364 responders for analysis (CARN group 147, random group 217). Under the assumption that nonresponders had the same percentage of foreign obstetrician-gynecologists (4%) and physicians who did not see obstetric patients as the responders (20%), the adjusted response rate for the CARN and randomly selected groups combined was 47% (364/768). Not all respondents answered every question.

**Table 1** Demographic and practice-related characteristics of US obstetrician-gynecologists, 1999

| *Characteristic* | *CARN*[1] (n = *147*) | *Random* (n = *217*) | p *value** |
|---|---|---|---|
| Age (mean; years) | 45 | 45 | 0.84 |
| Year of completing residency (mean) | 1985 | 1986 | 0.20 |
| Gender (percentage of total) | | | 0.04 |
| Male | 51.0 | 61.8 | |
| Female | 49.0 | 38.2 | |
| Region of United States (percentage of total) | | | 0.81 |
| Northeast | 20.4 | 20.2 | |
| South | 31.7 | 36.2 | |
| Midwest | 24.6 | 21.6 | |
| West | 23.3 | 22.0 | |
| Practice type (percentage of total) | | | 0.40 |
| Solo | 16.3 | 21.4 | |
| Multi-specialty group | 15.6 | 12.1 | |
| University | 13.6 | 9.8 | |
| OB/GYN partnership or group | 46.9 | 46.5 | |
| HMO | 2.7 | 4.7 | |
| Military | 1.4 | 3.3 | |
| Other | 3.4 | 2.3 | |

CARN, Collaborative Ambulatory Research Network;
*Comparison of CARN and random groups, Student's *t* test for means, $\chi^2$ for categorical data

### Demographic and practice-related information

The mean age of respondents was 45 years for both the CARN and the random groups (Table 1). A higher proportion of respondents from the CARN group was female than from the random group (49% vs. 38%, $\chi^2 p = 0.04$). Otherwise, the CARN and random groups were similar by year of completing residency, region of the United States[8], and practice type (Table 1).

### Diagnosis and laboratory testing

Overall, 91% responded that a positive *Toxoplasma gondii* IgG test indicates that a woman has had toxoplasmosis but does not determine when she was infected (Table 2). Nearly half (48%) thought a positive IgM test meant that a woman had acquired toxoplasmosis within the past 3 months, 6% within the past 12 months, and 3% within the past 24 months. Only 12% indicated that a positive IgM test could represent a false–positive result, and only 11% of respondents were aware that the FDA had sent an advisory to physicians in 1997 stating that there were problems with some commercial *T. gondii* IgM test kits because of high false–positive rates. Most (94%) indicated that, if a test for IgM was positive, they would consult with a specialist (for example, in infectious diseases or perinatology). Overall, 7% of respondents indicated that they had diagnosed one or more case(s) of recently acquired infection with *T. gondii* in a pregnant woman in 1998 (Table 2).

### Clinical aspects

Most (87%) ACOG members were aware that maternal *T. gondii* infections are not usually symptomatic (Table 2). A majority (63%) responded that risk of congenital disease is highest when toxoplasmosis is contracted during the first trimester, and 62% of members indicated that congenital toxoplasmosis is less severe when contracted in the last trimester (CARN group 57%, random group 65%; $\chi^2$ $p < 0.05$). One per cent of respondents indicated that all women with positive IgG tests should be treated. However, 53% of respondents indicated that all women with a positive IgM test should be treated for toxoplasmosis (CARN group 47%, random group 57%; $\chi^2$ $p < 0.05$).

Data are not shown in Table 2 for the following median responses. Respondents indicated that a woman with acute toxoplasmosis has about a 25% chance of infecting her fetus (median response, same result for random and CARN groups). Overall, respondents indicated that the use of spiramycin in a woman with acute toxoplasmosis reduces her chance of infecting her fetus by 50% (median response, same result for random and CARN groups). Respondents also indicated that 70% (CARN group) to 75% (random group) of congenitally infected infants appear normal at birth (median responses).

**Table 2** Responses from obstetrician-gynecologists in the United States to a survey about toxoplasmosis, 1999

| | CARN yes or affirmative | | Random yes or affirmative | | Total yes or affirmative | | Total 95% CI |
|---|---|---|---|---|---|---|---|
| *Question or statement* | *N** | *Number (%)* | *N** | *Number (%)* | *N** | *Number (%)* | *(%)* |
| *Diagnosis and laboratory testing* | | | | | | | |
| A positive *T. gondii* IgG test indicates a woman has been infected for how long? | | | | | | | |
| 2 years | 143 | 0 (0.0) | 215 | 3 (1.4) | 358 | 3 (0.8) | 0.0, 2.4 |
| 5 years | 143 | 2 (1.4) | 215 | 3 (0.9) | 358 | 4 (1.1) | 0.0, 2.8 |
| 10 years | 143 | 6 (4.2) | 215 | 6 (2.8) | 358 | 12 (3.4) | 1.7, 5.8 |
| In the past, but cannot say when | 143 | 128 (89.5) | 215 | 198 (92.0) | 358 | 327 (91.3) | 87.9, 94.0 |
| Don't know/other | 143 | 11 (7.7) | 215 | 8 (3.7) | 358 | 19 (5.3) | 3.2, 8.2 |
| A positive *T. gondii* IgM test indicates a woman has been infected for how long?** | | | | | | | |
| Within 3 months | 147 | 64 (43.5) | 217 | 112 (51.6) | 364 | 176 (48.2) | 43.0, 53.6 |
| Within 12 months | 147 | 12 (8.2) | 217 | 11 (5.1) | 364 | 23 (6.3) | 4.1, 9.3 |
| Within 24 months | 147 | 3 (2.0) | 217 | 8 (3.7) | 364 | 11 (3.0) | 1.5, 5.3 |
| Recent, but cannot determine when | 147 | 70 (47.6) | 217 | 112 (51.6) | 364 | 182 (50.0) | 44.7, 55.3 |
| Don't know | 147 | 6 (4.1) | 217 | 4 (1.8) | 364 | 10 (2.7) | 1.3, 5.0 |
| A positive *T. gondii* IgM test could be a false–positive reaction | 147 | 16 (10.9) | 217 | 27 (12.4) | 364 | 43 (11.8) | 8.7, 15.6 |
| Aware FDA sent advisory to physicians about false–positive *T. gondii* IgM tests in 1997 | 147 | 20 (13.6) | 216 | 19 (8.7) | 363 | 39 (10.7) | 7.8, 14.4 |
| Would consult a specialist if a *T. gondii* IgM test returned positive | 108 | 104 (96.2) | 157 | 146 (93.0) | 265 | 250 (94.3) | 90.8, 96.8 |
| Diagnosed one or more acute cases of toxoplasmosis in 1998 | 135 | 13 (9.6) | 198 | 9 (4.5) | 333 | 22 (6.6) | 4.2, 9.8 |
| *Clinical aspects* | | | | | | | |
| Maternal *T. gondii* infections are not usually symptomatic | 147 | 123 (83.7) | 216 | 191 (88.4) | 363 | 314 (86.5) | 82.6, 89.8 |
| The risk of congenital disease is highest if toxoplasmosis occurs in the first trimester | 145 | 93 (64.1) | 217 | 136 (62.7) | 362 | 229 (63.3) | 58.1, 68.2 |
| Congenital disease is less severe if toxoplasmosis occurs in the last trimester† | 145 | 83 (57.2) | 216 | 141 (65.3) | 361 | 224 (62.0) | 56.8, 67.1 |
| All pregnant women with a positive *T. gondii* IgG test should be treated | 142 | 2 (1.4) | 215 | 3 (1.4) | 357 | 5 (1.4) | 0.5, 3.2 |
| All pregnant women with a positive *T. gondii* IgM test should be treated† | 141 | 66 (46.8) | 214 | 122 (57.0) | 355 | 188 (53.0) | 47.6, 58.3 |
| *Screening for toxoplasmosis* | | | | | | | |
| Opposed or strongly opposed to universal screening of pregnant women for acute *T. gondii* infection† | 141 | 99 (70.2) | 211 | 125 (59.2) | 352 | 224 (63.6) | 57.7, 68.0 |

*Continued over*

**Table 2** (Continued)

| Question or statement | CARN yes or affirmative N* | CARN yes or affirmative Number (%) | Random yes or affirmative N* | Random yes or affirmative Number (%) | Total yes or affirmative N* | Total yes or affirmative Number (%) | Total 95% CI (%) |
|---|---|---|---|---|---|---|---|
| When are pregnant women screened for toxoplasmosis in your practice?** | | | | | | | |
| If they are considered high-risk | 146 | 91 (62.3) | 211 | 145 (68.7) | 357 | 236 (66.1) | 60.9, 71.0 |
| At every visit | 146 | 2 (1.4) | 211 | 0 (0.0) | 357 | 2 (0.6) | 0.1, 2.0 |
| At the initial exam | 146 | 35 (24.0) | 211 | 48 (22.7) | 357 | 83 (23.2) | 19.0, 28.0 |
| If the patient asks questions | 146 | 55 (37.7) | 211 | 70 (33.2) | 357 | 125 (35.0) | 30.1, 40.2 |
| If the patient mentions symptoms | 146 | 67 (45.9) | 211 | 102 (48.3) | 357 | 169 (47.3) | 42.1, 52.7 |
| Never | 146 | 20 (13.7) | 211 | 23 (10.9) | 357 | 43 (12.0) | 8.9, 15.9 |
| *Prevention* | | | | | | | |
| In order to prevent toxoplasmosis it is helpful for women to:** | | | | | | | |
| Keep a cat completely outdoors | 145 | 83 (57.2) | 213 | 105 (49.3) | 358 | 188 (52.5) | 42.7, 57.8 |
| Wear gloves when changing litter | 145 | 109 (75.2) | 217 | 176 (81.1) | 362 | 285 (78.7) | 74.2, 82.8 |
| Wear gloves when gardening | 146 | 116 (79.5) | 216 | 162 (75.0) | 362 | 278 (76.8) | 72.1, 81.1 |
| Cover sand boxes | 145 | 111 (76.6) | 213 | 148 (69.5) | 358 | 259 (72.3) | 67.4, 76.9 |
| Wash hands after handling meat | 146 | 127 (87.0) | 216 | 185 (85.6) | 362 | 312 (86.2) | 82.2, 89.6 |
| Eat only well cooked meat | 146 | 133 (91.1) | 217 | 193 (88.9) | 363 | 326 (89.8) | 86.2, 92.7 |
| Not consume unpasteurized foods | 146 | 89 (61.0) | 217 | 144 (66.4) | 363 | 333 (64.2) | 59.0, 69.1 |
| How often do you counsel pregnant women about preventing acute toxoplasmosis?** | | | | | | | |
| At every visit | 145 | 3 (2.1) | 212 | 2 (0.9) | 357 | 5 (1.4) | 0.5, 3.2 |
| At the initial exam | 145 | 95 (65.5) | 212 | 139 (65.6) | 357 | 234 (65.5) | 60.4, 70.5 |
| If the patient asks questions | 145 | 64 (44.1) | 212 | 88 (41.5) | 357 | 152 (42.6) | 37.4, 47.9 |
| If the patient mentions she was ill | 145 | 11 (7.6) | 212 | 16 (7.5) | 357 | 27 (7.6) | 5.0, 10.8 |
| If I consider the patient to be at high risk | 145 | 49 (33.8) | 212 | 83 (39.2) | 357 | 132 (37.0) | 32.0, 42.2 |
| Never | 145 | 5 (3.4) | 212 | 6 (2.8) | 357 | 11 (3.1) | 1.6, 5.5 |
| Your counseling includes information about**: | | | | | | | |
| Eating undercooked foods | 140 | 117 (83.6) | 207 | 172 (83.1) | 347 | 289 (83.3) | 78.9, 87.1 |
| Handling raw foods | 140 | 106 (75.7) | 204 | 157 (77.0) | 344 | 263 (76.5) | 71.6, 80.8 |
| Handling cat litter | 141 | 141 (100.0) | 207 | 207 (100.0) | 348 | 348 (100.0) | 99.0, 100.0 |
| Inadvertent contact with cat feces | 140 | 133 (95.0) | 203 | 198 (97.5) | 343 | 331 (96.5) | 94.0, 98.2 |
| Gardening | 139 | 100 (71.9) | 201 | 130 (64.7) | 340 | 230 (67.6) | 62.4, 72.6 |
| How do you provide toxoplasmosis-related information to pregnant women?† | | | | | | | |
| Verbally | 144 | 140 (97.2) | 215 | 205 (95.3) | 359 | 345 (96.1) | 93.5, 97.9 |
| Pamphlet | 144 | 14 (9.7) | 216 | 26 (12.0) | 360 | 40 (11.1) | 8.1, 14.8 |
| Other | 144 | 1 (0.7) | 216 | 5 (2.3) | 360 | 6 (1.7) | 0.6, 3.6 |

CARN, Collaborative Ambulatory Research Network; 95% CI, 95% confidence interval; *N varies owing to different response rates for each question; **respondents could check multiple answers for this questions; †CARN response differs from random group response, $\chi^2$ $p < 0.05$

### Screening for toxoplasmosis

A majority (64%) of ACOG members were opposed or strongly opposed to universal screening of pregnant women for toxoplasmosis (CARN group 70%, random group 59%; $\chi^2$ $p < 0.05$; Table 2). When asked how often pregnant women in their practice are screened for infection with *T. gondii* 66% of ACOG members responded 'when they are considered high risk', 1% responded 'at every visit', 23% responded 'at the initial exam', 35% responded 'if the patient asks questions', 47% responded 'if the patient mentions they have/had suggestive symptoms', and 12% responded 'never'.

The following data for open-ended responses are included here for completeness but are not shown in Table 2. Open-ended responses were collected to the question 'what patient characteristics would lead you to consider that a woman is at high risk for toxoplasmosis?' Among these open-ended responses about risk, 67% (CARN group 66%, random group 68%) of participants specifically mentioned cat exposure, 12% (11% CARN group, 13% random group) mentioned cat-litter exposure, 30% (32% CARN group, 30% random group) raw or undercooked meat exposure, and 9% (12% CARN group, 7% random group) gardening. Other less frequent responses included doing veterinary work, living on a farm, working with animals, living in a rural area, working in a restaurant, hunting, abnormal fetal ultrasound, fever/systemic illness, and history of living in a foreign country.

In addition, open-ended responses were recorded to a question that asked about the major concerns ACOG members had with screening pregnant women for toxoplasmosis. The most frequent concerns were that screening would be very costly and not cost-effective (44% overall; 44% CARN group, 43% random group), that with screening there would be many false–positive results or test results that are hard to interpret (29% overall; 29% CARN group, 30% random group), and that screening would create unnecessary anxiety in patients (17% overall; 17% CARN group, 17% random group). Some physicians (6% overall; 5% CARN group, 7% random group) thought that screening would lead to over-treatment or that treatment is not effective anyway (3% overall; 3% CARN group, 3% random group). When we asked about the cost of fully screening a woman for toxoplasmosis, the median response was $60 overall for an office visit (CARN group $50, random group $70), and for laboratory testing costs the median response was $100 for both the CARN and random groups.

### Prevention

Overall, ACOG members were well-informed about meat-, cat- and soil-related measures to prevent transmission of toxoplasmosis and indicated that they counsel women about these measures (Table 2). Many respondents indicated that they counsel pregnant women about toxoplasmosis at the initial examination (66%), when the patient asks questions about toxoplasmosis (43%), or when they consider the patient at high risk for toxoplasmosis (37%). Nearly all (96%) of the ACOG members surveyed indicated that they provide toxoplasmosis-related prevention information verbally, 11% indicated that they provide pamphlets, and 2% indicated they use other methods.

### DISCUSSION

In this survey of US obstetrician-gynecologists, we found that 7% of respondents had diagnosed one or more case(s) of recently acquired toxoplasmosis in a pregnant woman over a 1-year period. These results imply that a considerable proportion of obstetrician-gynecologists will encounter acute toxoplasmosis in a pregnant woman during their careers. Because treatment can help prevent severe consequences of congenital infection, it is important that obstetrician-gynecologists stay informed about the diagnostic and clinical aspects of toxoplasmosis.

Most respondents were aware that a positive *T. gondii* IgG test indicates that a woman could have been infected at any time in the past and that the IgG test is not by itself an indication for treatment. However, there was not a consistent understanding among obstetrician-gynecologists about the meaning of a positive *T. gondii* IgM test in the diagnosis of acute toxoplasmosis. Because numerous commercial *T. gondii* IgM tests have become available over the past 15 years, with various sensitivity and specificity rates, interpretation of results can be confusing. The largest problem has been false–positive results with some of the commercial IgM tests[9,10]. Because of the problems with false–positive *T. gondii* IgM test results, the FDA sent an advisory to physicians in the United States in 1997 calling attention to the limitations of these tests. Most obstetrician-gynecologists surveyed were not aware of this advisory. The *T. gondii* IgM test alone should not be used as a screening test for acute *T. gondii* infection. The *T. gondii* IgG test should be used first, because 86% of women of child-bearing age do not have evidence of prior exposure to *T. gondii*[11], and the IgG test can be used to rule out infection for most women. When the IgG test is positive, an IgM test should be ordered to determine whether the infection is relatively recent. If the IgM test is negative, then no further testing is required. If the IgM test is also positive, the woman may have been infected within the past 18 months. In this case additional testing should be done at a reference laboratory to rule out a false–positive IgM test and to determine whether the infection is likely to have occurred during the pregnancy[10]. Incorrect interpretation of false–positive *T. gondii* IgM test results could lead to inappropriate use of antiparasitic medication in pregnant women and possibly unnecessary interruption of pregnancy.

With regard to clinical symptoms of toxoplasmosis, most of the respondents were aware that maternal *T. gondii* infections are not usually symptomatic. Studies indicate that mild symptoms such as lymphadenopathy, fever and fatigue occur in 10–20% of immunocompetent adults and generally resolve without treatment in weeks to months[12]. However, there was less certainty among the obstetrician-gynecologists surveyed about the risk of congenital disease relative to the trimester when a woman acquires an infection with *T. gondii*. The risk of congenital infection is lowest (10–25%) when acute maternal infection occurs during the first trimester and highest (60% or higher) when acute maternal infection occurs during the third trimester[13]. However, the severity of disease is worse when infection is acquired in the first trimester[13,14]. The overall risk of congenital infection from acute *T. gondii* infection during pregnancy is 20–50%[13].

Respondents tended to indicate that treatment of acute *T. gondii* infection during pregnancy with spiramycin reduces the chance of congenital infection by about 50%. Some researchers have found that treatment during pregnancy reduces the chance of congenital infection by up to 60%[15–18]. However, others have stated that, because there were no directly comparable control groups in studies of treated women that found a reduction in congenital infection, it is unclear whether prenatal treatment in women with toxoplasmosis reduces congenital transmission of *T. gondii*[19]. Respondents also tended to indicate that most congenitally infected infants appear normal at birth, even though many will develop clinical problems later if untreated. This response is consistent with the literature[2,20–22].

A majority of the obstetrician-gynecologists surveyed were opposed to universal screening of pregnant women in the United States for *T. gondii* infection. However, from the survey there are no consistent criteria that emerge for when obstetrician-gynecologists should test pregnant women for acute *T. gondii* infection. Some respondents indicated that they test women at the initial prenatal examination; others indicated that they test a woman when she asks questions about toxoplasmosis or is considered to be at risk for acute infection. Interestingly, very few respondents (12%) indicated that they never test pregnant women for acute *T. gondii* infection.

The obstetricians surveyed gave a median response of a $60 cost for an office visit and a $100 cose for laboratory testing to fully screen a woman for toxoplasmosis. We have found that costs range from $50 to $100 each for IgG and IgM testing at commercial laboratories and that a complete confirmatory testing evaluation at a reference laboratory costs $300–400.

The obstetrician-gynecologists surveyed were well-informed about measures to prevent transmission of *T. gondii* to pregnant women. This result is particularly encouraging insofar as several studies, although not carried out in the United States, have shown that primary prevention messages are associated with improvement in *Toxoplasma*-related health behaviors[23] and a reduction in the rates of acute *T. gondii* infection during pregnancy compared to historic controls[24–26]. Nearly all ACOG members responded that they provide verbal counseling to pregnant women, although 11% indicated that they also provide pamphlets.

One area in which there was a wider range of responses from those surveyed had to do with care of cats. Always keeping a cat indoors greatly reduces the chances that a cat will acquire *T. gondii* infection because the cat is less likely to ingest prey that is infected with *T. gondii.* Changing the cat litter box daily helps prevent transmission of *T. gondii,* because, even under the best conditions (warm, moist environment), the oocysts excreted by cats take 1–5 days to sporulate and become infectious[27].

A limitation of the study is the low (38%) response rate among the random group. However, the response rate was much better in the CARN group (73%), and responses to most questions were very similar for the two groups. Nevertheless, response to the question 'should all pregnant women with a positive IgM test be treated?' was higher for the random group (57%) than for the CARN group (47%), and response to the question about being 'opposed to universal screening of pregnant women for acute *T. gondii* infection' was higher for the CARN group (70%) than for the random group (59%). The responses to these two questions may indicate that persons in the CARN group are more knowledgeable about the pitfalls of false–positive *Toxoplasma* IgM tests and more concerned about the possible adverse consequences of universal screening. Because persons in the CARN group volunteer to participate, they may be more knowledgeable about these issues. Non-response bias could also explain the differences.

Overall, it is apparent from our survey that a considerable proportion of ACOG obstetrician-gynecologists in the United States will encounter acute infection with *T. gondii* in their practice. Continuing education materials are being developed by ACOG and CDC based on this survey. Future research should focus on how effective continuing education materials are at improving knowledge about toxoplasmosis.

### REFERENCES

1. Mead PS, Slutsker L, Dietz V, *et al.* Food-related illness and death in the United States. *Emerg Infect Dis* 1999;5:607–25
2. Guerina NG, Hsu HW, Meissner HC, *et al.* Neonatal serologic screening and early treatment for congenital *Toxoplasma gondii* infection. *N Engl J Med* 1994;330:1858–63
3. Wong SY, Remington JS. Toxoplasmosis in pregnancy. *Clin Infect Dis* 1994;18:853–61
4. Lopez A, Dietz VJ, Wilson M, *et al.* Preventing congenital toxoplasmosis. *MMWR* 2000;49:57–75
5. StatXact Ver 4.01. Cambridge, MA: Cytel Software Corp.,1999
6. EpiInfo, Version 6. 1994. A word processing, database, and statistics system for epidemiology on microcomputers. Atlanta: CDC, 1994
7. SAS Version 6. Cary, NC: SAS Institute Inc., 1990
8. US Bureau of the Census. 2000. See http://www.census.gov/population/estimates/state/st-99-3.txt
9. Wilson M, Remington JS, Clavet C, *et al.* Evaluation of six commercial kits for detection of human immunoglobulin M antibodies to *Toxoplasma gondii. J Clin Microbiol* 1997;35:3112–15
10. Wilson M, McAuley JM. Toxoplasma. In Murray PR, Baron EJ, Pfaller MA, eds. *Manual of Clinical Microbiology*, 7th edn. Washington, DC: American Society for Microbiology, 1999
11. Wilson M, Lewis B, Fried J, *et al.* Seroepidemiology of toxoplasmosis in the United States. Abstract C545, 94th Meeting of the American Society of Microbiology, Las Vegas, NV, 1994
12. Remington JS. Toxoplasmosis in the adult. *Bull NY Acad Med* 1974;50:211–27
13. Remington J, McLeod R, Desmonts G. Toxoplasmosis. In Remington J, Llein J, eds. *Infectious Diseases of the Fetus and Newborn Infant*, 4th edn. Philadelphia: W.B. Saunders, 1994
14. Holliman RE. Congenital toxoplasmosis: prevention, screening, and treatment. *J Hosp Infect* 1995;30(Suppl):179–90
15. Desmonts G, Courvreur J. Congenital toxoplasmosis. A prospective study of 378 pregnancies. *N Engl J Med* 1974;290:1110–16

16. Desmonts G, Courvreur J. Congenital toxoplasmosis. A prospective study of the offspring of 542 women who acquired toxoplasmosis during pregnancy. In Thalhammer O, Pollak A, Baumgarten K, eds. *Pathophysiology of Congenital Disease* (6th European Congress of Perinatal Medicine, Vienna, Austria). Stuttgart: Georg Thieme, 1979:51–60
17. Forestler F. Les foetopathies infectieuses—prevention, diagnostic prenatal, attitude pratique. *Presse Med* 1991;20:1448–54
18. Hohlfield P, Daffos F, Thulliez P, *et al*. Outcome of pregnancy and infant follow-up after in utero treatment. *J Pediatr* 1989;115:767
19. Wallon M, Liou C, Garner P, Peyron F. Congenital toxoplasmosis: systematic review of evidence of efficacy of treatment in pregnancy. *Br Med J* 1999;318:1511–14
20. Carter AO, Frank JW. Congenital toxoplasmosis: epidemiologic features and control. *Can Med Assoc J* 1986;135:618–23
21. Wilson CB, Remington JS, Stagno S, Reynolds DW. Development of adverse sequelae in children born with subclinical congenital *Toxoplasma* infection. *Pediatrics* 1980;66:767–74
22. McAuley J, Boyer KM, Patel D, *et al*. Early and longitudinal evaluations of treated infants and children and untreated historical patients with congenital toxoplasmosis: the Chicago collaborative treatment trial. *Clin Infect Dis* 1994; 18:38–72
23. Carter AO, Gelmon SB, Wells GA, Toepell AP. The effectiveness of a prenatal education programme for the prevention of congenital toxoplasmosis. *Epidemiol Infect* 1989;103:539–45
24. Foulon W, Naessens A, Lauwers S, *et al*. Impact of primary prevention on the incidence of toxoplasmosis during pregnancy. *Obstet Gynecol* 1988;72:363–6
25. Foulon W, Naessens A, Derde MP. Evaluation of the possibilities for preventing congenital toxoplasmosis. *Am J Perinatol* 1994;11:57–62
26. Henri T, Jacques S, Rene L. Twenty-two years screening for toxoplasmosis in pregnancy: Liege-Belgium. *Scand J Infect Dis Suppl* 1992;84: 84–5
27. Dubey JP, Lindsay DS, Speer CA. Structures of *Toxoplasma gondii* tachyzoites, bradyzoites, and sporozoites and biology and development of tissue cysts. *Clin Microbiol Rev* 1998;11:267–99

Received 9/12/00; Accepted 11/09/00
